# The Application of Arbuscular Mycorrhizal Fungi as Microbial Biostimulant, Sustainable Approaches in Modern Agriculture

**DOI:** 10.3390/plants12173101

**Published:** 2023-08-29

**Authors:** Wenli Sun, Mohamad Hesam Shahrajabian

**Affiliations:** Biotechnology Research Institute, Chinese Academy of Agricultural Sciences, Beijing 100081, China; hesamshahrajabian@gmail.com

**Keywords:** arbuscular mycorrhizal fungi, biostimulant, microbial biostimulants, mycorrhizal fungi, sustainable agriculture

## Abstract

Biostimulant application can be considered an effective, practical, and sustainable nutritional crop supplementation and may lessen the environmental problems related to excessive fertilization. Biostimulants provide beneficial properties to plants by increasing plant metabolism, which promotes crop yield and improves the quality of crops; protecting plants against environmental stresses such as water shortage, soil salinization, and exposure to sub-optimal growth temperatures; and promoting plant growth via higher nutrient uptake. Other important benefits include promoting soil enzymatic and microbial activities, changing the architecture of roots, increasing the solubility and mobility of micronutrients, and enhancing the fertility of the soil, predominantly by nurturing the development of complementary soil microbes. Biostimulants are classified as microbial, such as arbuscular mycorrhizae fungi (AMF), plant-growth-promoting rhizobacteria (PGPR), non-pathogenic fungi, protozoa, and nematodes, or non-microbial, such as seaweed extract, phosphite, humic acid, other inorganic salts, chitin and chitosan derivatives, protein hydrolysates and free amino acids, and complex organic materials. Arbuscular mycorrhizal fungi are among the most prominent microbial biostimulants and have an important role in cultivating better, healthier, and more functional foods in sustainable agriculture. AMF assist plant nutrient and water acquisition; enhance plant stress tolerance against salinity, drought, and heavy metals; and reduce soil erosion. AMF are proven to be a sustainable and environmentally friendly source of crop supplements. The current manuscript gives many examples of the potential of biostimulants for the production of different crops. However, further studies are needed to better understand the effectiveness of different biostimulants in sustainable agriculture. The review focuses on how AMF application can overcome nutrient limitations typical of organic systems by improving nutrient availability, uptake, and assimilation, consequently reducing the gap between organic and conventional yields. The aim of this literature review is to survey the impacts of AMF by presenting case studies and successful paradigms in different crops as well as introducing the main mechanisms of action of the different biostimulant products.

## 1. Introduction

Biostimulant application is known as an eco-friendly and novel farming practice and is relevant to two otherwise contrasting concepts, namely crop sustainability and intensification [1]. Biostimulant products already form a significant part of the global farming industry, indicating increasing trends over the years and in the future [2]. There are various reports regarding their positive impacts on crops, especially under biotic and abiotic stress conditions, and significant research is continuously conducted to find and/or produce new biostimulatory products, as well as to show the mechanisms of action behind the observed impacts. However, the variance in the composition of biostimulant products, as well as the lack of ordinary application protocols for the different products, may create inconsistencies between the observed results and complicate attempts to reveal the actual mechanisms behind the biostimulatory impacts, which may include physiological procedures, hormonal regulation, and morphological alterations. Biostimulants’ beneficial activities include the improvement of nutrient uptake, the induction of root growth, and the production of phytohormones; osmotic adjustment through the synthesis of organic osmolytes has also been confirmed. Biostimulants can also be applied to decrease the application of mineral inorganic fertilizer and are considered environmentally friendly tools with no significant negative impacts on fruit quality or total yield. Humic acids, fulvic acid, protein seaweed extracts, hydrolysates, *N*-containing compounds, botanicals, seaweed extracts, chitosan and other related biopolymers, beneficial bacteria and fungi, and inorganic compounds are the major categories of plant biostimulants. Modern crop production has to cope with abiotic and biotic stressors such as soil and irrigation water salinity, extreme and untimely weather phenomena, water limitations, infections from pathogens, and pests, which severely influence crop performance and the quality of the final products [3,4,5]. The most important advantages of biostimulants include improved profits, stimulated plant reactions, decreased operating costs, reduced application of fertilizers, improved root protection from soil pathogens, and enhanced drought tolerance; moreover, they repel pests, accelerate root establishment, boost fertilization, enhance stress tolerance, ameliorate fertilization, alleviate leaching, detoxify heavy metals and chemicals, and improve stomata opening and plant transpiration [3,4,5,6]. Biostimulatory compounds may also have positive effects on soil biology and are recognized as a good technique for recovering semi-arid areas and degraded ecosystems [6,7,8]. However, the variable composition of raw materials applied for the production of biostimulant products makes the task of revealing the mechanisms of action more difficult, and long-term research and standardization processes are needed [9]. Different sources of chitin and chitosan in nature are crustaceans (lobster, shrimp, king crab), fungi (*Mucor rouxii*, *Penicillium chrysogenum*, *Aspergillus niger*, *Lactarius vellereus*), insects (ladybug, wax worm, silk worm, butterfly), and mollusks (shell oysters, squid pen). Crustacean shells are the most notable chitin source, and chitin recovery involves three steps consisting of demineralization, deproteination, and the elimination of pigments and lipids [10,11,12]. Microbial proteases such as *Lactobacillus* sp., *Bacillus* sp., *Pseudomonas* sp., *Serrati marcescens*, etc., are the most significant strains applied in chitin and chitosan production [10]. 

Biostimulants containing organic substances, humic acids, amino acids, algae extracts, and carbon and boron increased plant growth, yield, and shelf life of onion bulbs [13], and the application of diluted honey extract (DHE) improved photosynthetic parameters, antioxidant activity, biomass production, and yield [14]. The use of seaweed extracts, vermicompost, and a mixture of animal waste increased yield and bulb traits [15]. Foliar application of vermicompost leachate, smoke-water, *Ecklonia maxima* extracts, and indole-3-butyric acid on seedlings of mustard greens grown in soils from goldmines boosted phytoremediation activities through the accumulation of heavy metals [16]. Foliar application of Kelpak SL and Asahi SL increased the nutritional value and improved the storage life of carrots [17], while root and foliar application of protein hydrolysates in lettuce plants grown under salinity conditions mitigated oxidative stress and increased glucosinolate and osmolyte content [18,19]. In intensive cropping sectors such as horticulture and floriculture, biostimulants can also boost nutrient use efficiency, partly substitute chemical fertilizer inputs, and ameliorate the quality and yield of crops [20,21]. Biostimulants based on microorganisms are a subgroup of the heterogeneous family of biostimulants, related to a microorganism (or mix of microorganisms) that can stimulate biochemical and physiological processes that benefit the nutrient efficiency, nutrient uptake, abiotic stress tolerance, crop quality, and/or yield of plants [22], which can moderately mitigate the damaging effects of intensive agriculture [23,24,25]. The most common microorganisms included in this group of biostimulatory products are the non-pathogenic and non-toxigenic bacteria of *Azotobacter* spp., *Rhizobium* spp., and *Azospirillum* spp., as well as different mycorrhizal fungi [24]. Mycorrhizas are a symbiotic association between fungi and plant roots and are present in several forms according to the fungal taxonomy and the host plant. Two important parameters that influence the distribution of these forms are the climatic and soil conditions and the host plant distribution [26,27] (Hart and Reader, 2002; Yang et al., 2012), and mycorrhiza can significantly boost the efficiency of mineral absorption, falling into two major categories: endotrophic and ectotrophic [28]. The main types of arbuscular mycorrhizal fungi (AMF) are related to the sub-phylum Glomeromycotina of the phylum Mucoromycota [29], and four orders of AMF, namely *Glomerales*, *Paraglomerales*, *Archaeosporales*, and *Diversisporales*, have been recognized in this sub-phylum, which contains 25 genera [30,31]. The protective mechanisms are credited to arbuscular mycorrhizal fungi-assisted alleviation of oxidative stress, rapid water uptake and nutrient absorption, and changes in the transcript levels of genes involved in signaling pathways or stress response [32,33,34], and the effectiveness of AMF is usually influenced by environmental variables and soil conditions [35]. Considering the numerous literature reports in the past decade related to microbial biostimulants and their impacts on different crops, this review aims to present the most up-to-date key results of microbial biostimulant practical applications on crops and the new tools available to unravel the mechanisms behind the observed impacts. In the present review, all relevant reports in the English language were collected. The literature search was performed by using the keywords of plant biostimulants, microbial biostimulants, arbuscular mycorrhizal fungi, mycorrhizal fungi, and sustainable agriculture in the main indexing systems, including PubMed/MEDLINE, Scopus, the Google Scholar search engine, as well as the Institute for Scientific Information Web of Science, from July 2000 to July 2023.

## 2. Biostimulant Categories

Biostimulants are classified into two distinct groups based on their origin; one category includes products that have biological origins in pathogens or plants, and the second group consists of products that do not have biological origins [36,37,38]. Another classification approach divides biostimulant products into microbial biostimulants, which are obtained from arbuscular mycorrhizal fungi and plant-growth-promoting bacteria, and non-microbial biostimulants, which include plant micro-algae extracts, humic substances, and biopolymers such as chitosan [39,40,41,42,43]. Different compounds with bioactive properties can be used as biostimulants to boost plant growth and development under normal and stress conditions [44,45,46,47,48,49,50,51,52]. Salicylic acid is economical and quick in action, environmentally sound, and it also links with other elicitors to boost the biosynthesis of secondary metabolites [53,54,55]. Humic acid can increase plant growth, retain water, enrich nutrients, and suppress disease [56,57]. Fulvic acids are used in sustainable horticulture and can change plant primary and secondary metabolism and increase nutrient uptake, root growth, and crop tolerance to environmental stresses [58,59]. Protein hydrolysate biostimulants, mostly produced by chemical and enzymatic hydrolysis of plant- and animal-derived proteins, are based on a mixture of soluble amino acids and peptides and can increase the yield and quality of products as well as improve the nutrient uptake and abiotic stress tolerance of plants [60,61,62,63]. They are largely prepared from brown seaweeds, such as *Ecklonia maxima*, *Ascophyllum nodosum*, and *Macrocystis pyrifera*, and they include promoting hormones or trace elements such as Zn, Fe, Mn, and Cu [64,65]. Humic-like substances such as fulvic and humic acids may also show biostimulatory activity, since several reports have suggested improved crop performance attributed mainly to auxin- and cytokinin-like impacts; they are obtained from organic matter decomposition and metabolic products of soil microbes, and they have roles in plant growth via the improvement of soil physical–chemical properties and the boosted availability of nutrients in the rhizosphere [66,67,68]. The actual mechanisms of action seem to be the result of synergy between the several bioactive components in raw materials, although the impacts may change depending on the crop, soil type, and soil microbes present in the rhizosphere [69,70,71,72]. The most important impacts of chitin and its derivatives’ applications are that they stimulate and protect seed germination, stimulate stress resistance, mitigate negative impacts of abiotic stress, induce plant growth and development, improve soil properties and prevent nutrient leaching, improve the shelf-life of crops, chelate heavy metals, increase crop yield and quality, and protect against pests and pathogens, e.g., bacteria, viruses, fungi, insects, and nematodes [73,74,75]. Amino acids are the best candidates to boost stress tolerance through osmo-protection, ROS scavenging, metal chelation, and nutrient availability [76], which can notably impact the synthesis and stimulation of some enzymes and gene expression [77,78,79]. They can also be applied as signal molecules, like for inducing stomatal closure, as sensors of the nutrient contents of cells, or as regulators for inducing their own catabolism. Amino acids can manage the procedure of protein synthesis, strengthening plant growth, photosynthesis, and yield formation. They can increase nutrient assimilation, use, and translocation, as well as increase the quality of constituents [79]. Amino acids are well-known biostimulants due to their positive effects on yield and plant growth, and can mitigate injuries from abiotic stresses [80,81]. Amino acids also have a significant role in ammonium fixation and C_4_ metabolism and in the biosynthesis of different components, including isoflavonoids, flavonoids, cutin, aurones, sporopollenin, stilbenes, proanthocyanidins, suberin, lignins, catechins, phenylpropenes, lignans, acylated polyamines, and other different alkaloid derivatives. The largest and most diverse group of secondary metabolites in plants is phenols, which have good antioxidant effects and are involved in the regulation of photosynthesis, physiological activities, oxidation reduction procedures, and plant breathing [82]. Phenolic acids and their derivatives are coumarins, stilbenes, quinones, lignans, flavonoids, curcuminoids, and tannins, which have meaningful roles in plant development, especially in pigment and lignin biosynthesis, and of course, they have a significant role in protecting plants from stress [83]. 

**Figure 1:**
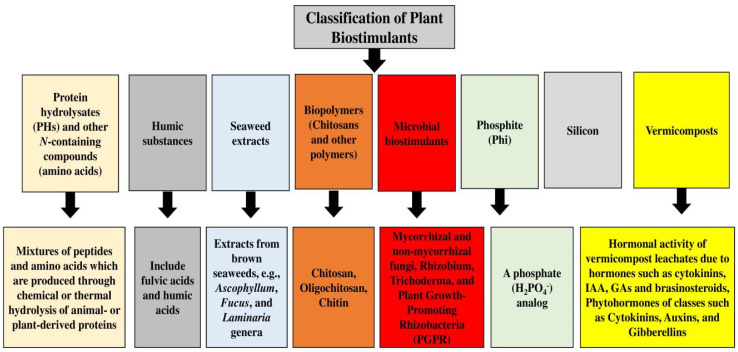
Different classifications of plant biostimulants.

Protein hydrolysate biostimulants, mostly produced by enzymatic and chemical hydrolysis of plant-derived and animal proteins, are based on a mixture of peptides and soluble amino acids, and can increase the quality and yield of products as well as the uptake and abiotic stress tolerance of plants [84]. *Glomus*, the largest and most common genus in the phylum *Glomeromycota*, forms symbiotic relationships with plant roots [85,86], which can boost the drought tolerance of the host plant, mediated by proteins with chaperone-like activity [87]. *Trichoderma* fungi have important functions in nature as plant growth promoters and antagonists of phytopathogenic fungi [88], and as rhizosphere inhabitants, they contribute to interactions with microorganisms, soil, arthropods, and plants at multiple trophic levels [89], and can be used as biocontrol and biopesticide agents [90]. Members of the genus *Trichoderma* are also used in different industry branches, like in the production of biofuel, antibiotics, and enzymes [91]. The main *Trichoderma*–plant interactions include their impacts on plant morphology, plant physiology, nutrient absorption and solubilization, disease resistance, yield improvement, and abiotic stress tolerance [92]. *Trichoderma reesei* is a genus of filamentous fungi and a superior cellulose source for industrial uses, and it can produce proteins, including different enzymes, cellulases, hemicellulases, and hydrophobins [93,94]. The endophytic fungus *Heteroconium chaetospira* can also penetrate through the outer epidermal cells of its host, pass into the inner cortex, and grow all over the cortical cells, consisting of those of the root tip region, without causing apparent pathogenic symptoms [95], and it can provide even more nitrogen to the plant than mineralizing plant-available organic nitrogen [96]. *Arthrobacter* species, which are Gram-positive chemoorganotrophs and obligate aerobes, are commonly identified among soil bacteria [97], being dominant aerobic bacteria under the class of families *Micrococcaceae* and *Actinobacteria* [98], and nutritional versatility is the principal feature of arthrobacters [99]. *Acinetobacter* spp. are Gram-negative coccobacilli that are aerobic, non-motile, and oxidative negative, with no glucose fermentation ability; they can be found in different environments [100] and can fix nitrogen, solubilize minerals, produce siderophores, and act as plant endophytes or epiphytes, which can help hosts in detaching pollutants and tolerating environmental stresses [101]. Moreover, the plant-growth-promoting traits of *Actinobacteria* entail phosphate solubilization, IAA, and siderophores [102]. They can also promote higher phosphorus content and plant growth and increase radical scavenging, plant phenolic components, and antioxidant activity [103]. Other important bacteria are *Enterobacter* spp., *Pseudomonas* spp., *Ochrobactrum* spp., *Bacilus* spp., and *Rhodococcus* spp. [104,105,106,107,108,109,110,111,112,113]. Figure 1 shows different classifications of plant biostimulants. 

## 3. Microbial Biostimulants

There are two kinds of microbial inoculants: biopesticides and biofertilizers. Biofertilizers are categorized under biostimulants [114,115]. They are also known as bioinoculants, which consist of living organisms and promote plant growth via a variety of procedures, such as increasing biomass and root growth, supplying nutrients, and increasing the capacity of nutrient uptake when applied to plants, seeds, or soil [116]. Microbial biostimulants have numerous positive functions associated with the solubilization, uptake, primary and secondary metabolism, and translocation of macro- and micro-nutrients, which induce phytochemical accumulation, the development of robust root systems for foraging surrounding soil, and the improvement of photosynthetic activity to promote growth, increase nutrient use efficiency, and stimulate antioxidant defense systems to decrease the oxidative stress burden [117]. Recently, biostimulants related to living microorganisms have attracted the attention of both academics and industry professionals for the simple reason that the growth and development of a plant can be increased more easily in the field [118,119]. These microbes are effective enzyme producers and are potential alternatives to formulating beneficial microbial consortia that can be used in tandem with gelatin to increase biostimulant activity [120]. Microorganisms acting as biostimulants mostly belong to beneficial fungi groups consisting of arbuscular mycorrhizal fungi and free-living bacteria [116,121,122]. Many parameters are responsible for the development of microbial inoculants as biofertilizers, such as the variety of plants [123,124] and the compatibility with different types of soil, chemical fertilizers, and environmental conditions [123,124]. The activity of microbial inoculants is mainly affected by root exudates (extracellular secretions by plants) and they also act as a substrate for the formation of biologically active substances [125]. AMF play a significant role in stimulating plant growth via different mechanisms: (i) increasing the uptake of water, as AMF boost the surface area of the root so that the plant can easily take up water; (ii) modifications of root architecture; (iv) availability of nutrients, particularly phosphorus, under nutrient-deficient conditions; (v) alterations in enzymatic and physiological activities, especially for plants that are active in antioxidative responses; and (vi) induction of ABA plant hormones, which are mostly involved in stress conditions [126,127]. Polyhydroxyalkanoates (PHAs) are selected as a carbon source for abundant microbial primary degraders [128]. 

## 4. Mechanisms of Microbial Biostimulant Action

Various notable protective mechanisms in the utilization of microbial biostimulants under different stresses are osmolite production, phytohormone level modulation, increased antioxidant activity, and secretion of extracellular polymeric substances (EPS) under water stress such as flooding and drought [129,130,131]; phytohormone level modulation, emission of volatile organic components, ice nucleation activity antagonism, delay of senescence, and osmo and thermal protection under thermal stress such as freezing and extreme heat [132,133,134,135]; increased soil exploration and mineral nutrient solubilization under nutrient stress [136,137]; and induced system resistance, direct antagonism with pathogens, and phytohormone level modulation under biotic stress [138,139,140,141,142]. Bozhinova [143] reported that the application of the microbial inoculant Europlus^®^ and the protein hydrolysate (PH) Trainer^®^ increased the yield of tobacco by 5.5% and 6.7%, respectively, in comparison with untreated plants. Bozhinova [143] also noted that the application of the microbial inoculant Europlus boosted N, P, and K concentrations in leaves, and the concentrations of Ca, Mn, Zn, and Cu were slightly higher than the control treatment when the microbial inoculant Europlus was applied, and concluded that the use of microbial biostimulants in sustainable tobacco farming was effective in boosting the yield of oriental tobacco and the quality of cured leaves. AMF can increase dry weight, photosynthesis, and seed fresh yield of *Glycine max* under drought stress [144]. Under drought stress, *Gigaspora decipiens* and *Glomus mosseae* can boost the chlorophyll content and growth of *Triticum aestivum*, and *Rhizophagus intraradices* can improve levels of Mg, Zn, Cu, and F in grains of *Triticum durum* [145,146]. Under drought stress, *Rhizophagus intraradices* can increase N, P, and K uptake in *Zea mays* [147]; *Funneliformis geosporus* BEG11 can increase water use efficiency in *Fragaria ananassa* [148]; *Glomus deserticola* can improve the level of proline and the number of leaves in *Antirrhinum majus* [149]; and *Gigaspora gregaria* can increase mineral levels and decrease the level of proline in *Vigna subterranea* [150]. Amid water shortage, *Paraglomus occultum* can increase the rate of water absorption and the length of the hypha in *Poncirus trifoliata* [151]; *Rhizophagus irregularis* can increase the conductivity of stomata and dry matter of shoot in *Digitaria eriantha* [152]; and *Glomus* species can improve the osmotic potential adjustment in *Ipomoea batatas* [153] and increase water uptake and phenolic, metabolite, and glutathione levels in *Saccharum arundinaceum* [154]. Under salinity stress, *Rhizophagus irregularis* can increase the fresh weight of shoots and roots and the number of leaves of *Solanum lycopersicum* [155], *Claroideoglomus etunicatum* can increase the conductivity of stomata and the level of soluble sugars in *Aleurites moluccanus* [156], and *Claroideoglomus etunicatum* can boost the dry mass of shoots and roots as well as the conductivity of stomata in *Aeluropus littoralis* [156]. Under cold stress, *Rhizophagus irregularis* and *Funneliformis mosseae* can increase photochemical reactions, decrease the damage in the membrane, and activate the antioxidant defense system in *Solanum melongena* [157]. *Rhizophagus irregularis* can improve the plant photosynthetic efficiency of *Solanum lycopersicum* under heat stress [158]. AMF containing *Rhizoglomus irregulare* and *Funnerliformis mosseae* can significantly influence the growth, productivity, and nutraceutical and nutritional quality of tomato cultivars, as AMF increase the biosynthesis and nutrient uptake of notable molecules involved in cellular pH and oxidative stress [159]. Six single strains (*Acaulospora laevis*, *Acaulospora scrobiculata*, *Gigaspora gigantea*, *Entrophospora colombiana*, *Glomus manihotis*, and *Scutellospora heterogama)* and a mixture of AMF (*Glomuss mosseae*, *Glomus manihotis*, and *Glomus gigantea*) recultured with *Chloris gayana* increased survival, improved tolerance against stress, and boosted the nutritional status, relative water content, photosynthetic rate, and the contents of P, N, Mg, and Fe in *Vitis vinifera* cv. Pusa Navrang [160]. *Rhizophagus irregularis*, *Pseudomonas fluorescens*, and *Funneliformis mosseae* application lead to an increase in APX and GPX enzyme activities, an enhancement of plant growth parameters, the alleviation of water deficit damage, a decrease in H_2_O_2_ and lipid peroxidation, and an improvement of drought tolerance in *Cupressus arizonica* Green [161]. The utilization of *Glomus mosseae*, *Rhizobium leguminosarum*, and *Arthrobacter protophormiae* induced a decrease in proline content and lipid peroxidation, an improvement of plant weight, an enhancement of nutrient uptake, an increase in pigment content, and the alleviation of salt stress of *Pisum sativum* under salt stress [162]. The application of *Acaulospora* sp., *Claroideoglomus etunicatum*, *Rhizobium* sp., and *Burkholderia* sp. led to an improvement in the absorption of chemical fertilizers and an increase in the wood yield of *Schizolobium parahyba* var. *amazonicum* under salt stress [163]. *Bacillus megaterium*, *Rhizophagus irregularis*, and *Frateuria aurantia* induced an enhancement of nutrient uptake responses, an improvement of low-mobility nutrient uptake such as Zn and Ca, an enhancement of total microbial biomass and microbial metabolism, an increase in plant growth, and an increase in gluten quality of *Triticum aestivum* L. under salt stress [164]. Miceli et al. [165] reported that the application of microbial biostimulants altered seedling growth and responses to different stresses and had a growth-promoting impact on the unstressed seedlings, increasing dry and fresh biomass accumulation, leaf area, and leaf number, being an appropriate option for improving the salinity tolerance of seedlings, particularly under salinity stress. 

## 5. Case Studies and Practical Application of Microbial Biostimulants 

There are two types of mechanisms of action of microbial biostimulants. For direct mechanisms, it has been reported that microbes are active in the synthesis of components that can boost the adsorption of nutrients, while indirect mechanisms refer to siderophore production, zinc solubilization, phosphorus solubilization, indole acetic acid biosynthesis, antioxidant enzyme production, ammonia and hydrogen cyanide production, biological nitrogen fixation, and phytohormone production [166,167]. Microbial biostimulants can alleviate the adverse effects of environmental stresses by producing hormone-like stimulants with positive impacts on plant growth and final yield [168]. Molecular processes that have roles in the interactions of plants and microorganisms which may lead to the biosynthesis of secondary metabolites are the protective impacts of microbial biostimulants on plants against different stressors [169]. The production of protective molecules is obtained via the shikimate pathway which may consist of the enzyme phenylalanine ammonia lyase (PAL) for the production of phenylpropanoids after microbial eliciting [170], which may have significant effects to adjust the pressure from external factors, called induced systemic resistance (ISR) [171]. The main mechanisms targeted by microorganisms according to various biostimulants can target shoot targets such as stomatal regulation and xylem hydraulic conductance, and root targets such as root zone water availability, root ethylene and auxin levels via ROS scavenging, membrane stability, and osmoprotection [172]. Biostimulants can influence plant phenotype, cellular level, and molecular level. The impact on plant phenotype consists of improved shoot and root growth, improved flowering, higher yield parameters, higher leaf number and vigor, better abiotic stress tolerance, better fruit nutritional quality, increased relative water content, increased stomatal conductance, and higher nutrient acquisition uptake and transportation. The effects at the cellular level include increased antioxidant activity, improved primary and secondary metabolisms, increased chlorophyll content, and higher photosynthetic rates, and those at the molecular level entail increased gene expression of SOD, CAT, APX, nutrient transporters, and stress-related genes [173]. Microbial biostimulants can alleviate abiotic stress, balance plant hormone levels, regulate indole-3-acetic acid (IAA), regulate cytokinins, regulate gibberellins, regulate abscisic acid, produce ACC deaminase, facilitate nutrient availability, modify root biomass and morphology, induce antioxidant enzyme syntheses in crops, accumulate osmolytes in crops, induce gene resistance to drought and salt stress, improve crop organic and inorganic pollutant toxicity tolerance, mediate increased nutrient availability (N, P, K, Fe, and Zn), improve crop cold tolerance, increase crop heat tolerance, and boost crop waterlogging tolerance [174,175,176,177,178,179]. It has been reported that microbial-based biostimulants boosted plant yield, Cu and Ca, and isochlorogenic acid components, and the influence of biostimulants on the functional and nutritional quality of lettuce was mainly independent of water availability [180]. Microbial biostimulants can significantly improve carbohydrate, protein, sugar, K, Ca, Mn, Zn, and Fe content in chickpea seeds and plants, and the microbiome analysis indicated a positive correlation with soil health and yield [181]. Mrid et al. [182] reported that microbial-based biostimulants can positively influence cropping systems via numerous mechanisms and can increase nutrient uptake and use efficiencies, suppress phytopathogens infection, boost root system development, reduce heavy metal toxicity, and improve crop growth and yield. Natural biostimulants can increase tolerance against abiotic stresses in agricultural and horticultural crops [183,184] and increase crop productivity under environmental stress [185,186]. Alfonzetti et al. [187] showed the potency of microbial biostimulants to positively influence native plant emergence and growth, but the exact impacts are linked to the plant species, type of amendment, and the characteristics of the planting site soil. Microbial biostimulants can increase ornamental plant growth during production and increase crop performance under abiotic stress, and appropriate microbes can also produce secondary metabolites like volatile organic components to improve plant growth. Aside from the direct impacts on molecular procedures, the effects of microbial biostimulants are related to morphological alterations such as changes in root morphology after inoculation with AMF and the increase in root surface, which can both increase the uptake of nutrients and water, thus helping plants cope with the negative impacts of stressors [188]. It has also been reported that the inoculation of water-stressed plants with *Penicillium* sp., *Phoma glomerata*, *Glomus intraradices*, *Exophiala* sp., and *Paecilomyces formosus* may lead to greater soil exploration by roots or fungal hyphae with meaningful improved root conductivity [189,190].

## 6. Arbuscular Mycorrhizal Fungi (AMF)

AMF, favorable microorganisms extensively distributed in nature, can establish a symbiotic relationship with most terrestrial herbs. AMF belong to 11 families, 25 genera, and nearly 250 species [191]. Arbuscular mycorrhizal (AM) symbiosis is the widespread mutualistic relationship between fungi and plants and plays an important role in nutrient exchange, ecosystem sustainability, the enhancement of plant stress resistance, and the development of host plants [192,193,194,195,196]. AMF increase the C allocation of the host plant below ground, which can form a microbial community composition [197]. AMF belong to the phylum *Mucoromycota* and subphylum *Glomeromycotina*. AMF are available in our natural environment and are helpful in several ways; they play an important role in increasing plant nutrition acquisition, improving plant tolerance and resistance to stresses, and increasing soil fertility and structure, and have several beneficial applications in agriculture [198,199,200]. Symbiosis can increase host plant nutrition because plants can absorb nutrients not only through mycorrhizal pathways but also through their own pathways. Besides that, AMF can also influence nutrient availability by altering soil physicochemical characteristics, microbial communities, and nutrient cycling [201,202,203]. AMF have notable effects on herbaceous plant element stoichiometry, such as plant C:N:P stoichiometry, which depends on plant and fungal functional group identities and soil nutrient availability [204]. 

**Figure 2:**
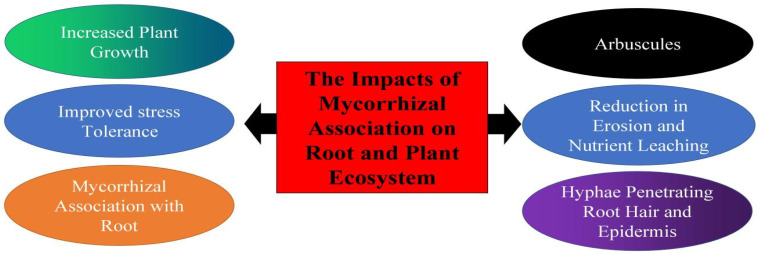
The influences of mycorrhizal association on root and plant ecosystem.

AMF promoted the utilization and accumulation of Ca in apple rootstock (*Malus robusta*) by adjusting the expression levels of genes associated with these pathways. GO and KEGG pathway analysis showed that gene expression changes in different critical gene families, such as auxin response (*MdGH3*, *MdAux/IAAs*, and *MdSAUR*), TCA cycles (*MdCS*, *MdMDH*, and *MdACO*), phosphate transporters (*MdPHT1;1*, *MdPHT1;10*, and *MdPHT1;3*), and Ca^2+^ signal transduction pathways (*MdCa^2+^/ATPase*, *MdTPC1*, *MdCML*, and *MdCDPK*), showing that apple stimulates the expression of genes related to auxin synthesis, organic acid secretion, and calcium transporters and channels, thus promoting the growth of apple root and improving the secretion of organic acids, which may lead to an increase in calcium effectiveness in soil [205]. Uptake of ZnO nanoparticles in barley was increased by AMF, possibly related to the mutualistic connection between the root and the fungi, which promotes water absorption because of the more efficient property of the rhizosphere [206]. The combination of humic substance and AMF remarkably increased plant biomass [207]. The impacts of *Rhizobium* and AMF inoculation were investigated on the growth and yield of *Lablab purpureus*, *Vigna unguiculata*, and *Mucuna pruriens*, and in comparison to single inoculation and uninoculated forages, the dual inoculation with AMF and *Rhizobia* improved the growth and biomass yield of forage legumes, and dual inoculation could be an optimal alternative in attempts to decrease the overreliance of chemical fertilizers on forage production [208]. The effects of mycorrhizal association on plant and root ecosystems are presented in Figure 2. 

When AMF are inoculated in a plant, they increase the plant’s tolerance against different stresses such as heavy metals, drought, and high temperatures. AMF form spores and hyphae in the rhizosphere, while inside the root tissues, they shape arbuscular, vesicles, and hyphae to improve the accessibility of plant roots to large soil surface areas by hyphal network knowledge with roots of plants, therefore increasing growth in the plant [209]. AMF synergistically improve plant P nutrition, and feedstock origin determines P accessibility and, as a consequence, mycorrhizal performance [210]. AMF inoculation may lead to the promotion of leaf chlorophyll content, shoot height, leaf photochemical efficiency, RWC, turf quality, and root and shoot biomass, as well as enhanced melatonin (MT) content through the upregulation of the MT biosynthesis genes and the reduction in the levels of malondialdehyde (MDA), electrolyte leakage (EL), chlorophyll catabolic genes (CCGs), hydrogen peroxide (H_2_O_2_), and senescence-associated genes (SAGs) to decrease heat stress in perennial ryegrass [211]. AMF inoculation increased gibberellin (GA), internal MT, and cytokinin (CTK) levels while reducing ABA levels in heat-stressed plants; moreover, the MT biosynthesis genes (*LpASMT1*, *LpTDC1*, *LpCOMT1*, *LpTDC2*, and *LpASMT3*), the abscisic acid (ABA) catabolic gene, the CTK biosynthesis genes, the GA biosynthesis genes, and the associated signal transduction response transcription factors (TFs) (type-B ARRs) showed increased levels, whereas the expression of the GA to ABA exerted decreased levels after MT application and AMF inoculation in heat-stressed plants [211]. AMF can increase earlier flowering of plants, promote fruit and flower production, prolong the total duration of flowering, and increase seed yield, showing potential in promoting seedling growth and seed germination [212]. 

AMF increased the plant height, root weight, root length, and stem weight of lettuce, and the functional composition profiles showed that several functions were increased, including cell motility and environmental adaptation, and AMF had a significant influence on the lettuce endophytic bacterial network community and structure function [213]. AMF with high Cu components provided adaptive mechanisms for the plant growth and survival of grapevine rootstock; in greenhouse conditions, stomatal conductance and transpiration improved with Cu additions, influencing plant growth [214]. AMF could inhibit the uptake of Hg, particularly methyl-Hg in grains of rice, as it may lead to Hg transfer in the non-edible parts of rice, such as the leaf and stem, and promote the growth indexes and micro-indexes of rice, and it can be a practical remediation technology for soil heavy metal pollution [215]. AMF also responds to Cd stress in kenaf; inoculation with AMF could be applied to kenaf to enhance the removal of Cd from soil in mining areas by phytoremediation, as AMF can adjust the expression levels of key kenaf genes, such as *Hc.AKR*, *Hc.GH3.1*, and *Hc.PHR1*, thus playing an important role in enhancing kenaf’s Cd tolerance [216]. 

Micro-proton-induced X-ray emission was utilized to determine element localization and indicated that AMF improved the nutrient uptake by wetland plants (*Iris pseudacorus*) and suppressed Cr translocation from roots to shoots, which showed that the interaction between plants and AMF could significantly improve the immobilization of high Cr concentrations in semi-aquatic habitats [217]. AMF inoculation and/or legume intercropping uphold the stability of microbial networks by weakening taxonomic interactions and improving modularity under drought events; overall, AMF inoculation causes *Medicago sativa* to need water during a drought event, which worsens soil water deficit and thus may increase interspecific water competition between *Medicago sativa* and *Broussonetia papyrifera*. The results of one experiment revealed that in the roots of tomato plants colonized by AMF, there was a meaningful mutual relationship between the insects feeding on the plant and the fungal species (*Rhizophagus intraradices*, *Funneliformis mosseae*, *R. irregularis*, and *Glomus iranicus*) [218]. 

AM fungi (*Glomus* spp.) play a role in upregulating plant growth and nutrition status in Fe-ore tailings in technosols of host plants like sorghum, providing the basis for the involvement of AM fungi in the eco-engineered pedogenesis of iron ore tailings [219]. In the ethanolic extracts of leaves of *Anadenanthera colubrina* (Vell.) Brenan, seedlings had more saponins and flavanols and showed significant antioxidant activity when inoculated with AMF (*Gigaspora albida* and *Acaulospora longula*) [220,221]. AMF significantly improved the biomass and nutrient levels of *Iris tectorum* and reduced the constituents of Cr in soils; furthermore, it can boost the abundance of functional genes related to nutrient cycling (N, P) in rhizosphere microbial communities, increase the abundance of functional genes associated with heavy metal resistance and transporters in the rhizosphere microbial community, and increase the complexity and stability of the rhizosphere microbial community [222]. AMF reduce the plant uptake and bioavailability of Cd and As, and the reduction in As and Cd accumulations induced by AMF depends on the plant family [223]. It has also been reported that AMF–*Glomus versiforme* (Gv) symbiosis significantly increased P uptake, plant growth, and photosynthesis in upland rice, and Gv inoculation decreased the expression of *Nramp5*, thus reducing Cd absorption, transfer, and accumulation in upland rice and enhancing the activities of catalase (CAT) and peroxide (POD) in Gv-inoculated upland rice [224]. *Glomeraceae* and *Paraglomeraceae* are the dominant fungi affecting the available P content. Expanding *Glomeraceae* and *Paraglomeraceae* and applying them to *Eucalyptus* plantations may increase soil P availability, and the structure of the AMF community may be a sensitive indicator of the quality of soil-available P in *Eucalyptus* plantations [225]. AMF reduced the toxicity risk of Pb by accumulating Pb in fungal structures, and both flooding and Pb stress reduced the AMF diversity but not the abundance [226]. 

Inoculation with indigenous AMF such as *Glomus* sp. and *Paraglomus* sp. boosted the biomass of plants and promoted root growth under replanting conditions, and it can also induce the activity of chitinase and β-1,3-glucanase and increase the resistance of apple rootstock by upregulating root reactive oxygen species levels and the antioxidant system under replanting conditions [227]. It is suggested that mycorrhizal fungi can reduce the detrimental impacts of salt stress on *Ligustrum vicaryi* plants, and the mediation of Ca^2+^, Zn^2+^, N, Mg^2+^, and soluble protein components could be the basic mechanism underlying salt tolerance in mycorrhizal plants [228]. AMF and root hairs are functionally redundant in maize, and AMF had a greater effect on microbial structure than root hairs, as well as a greater role in microbial P mineralization than root hairs of maize [229]. Activities of 4-coumaroyl-CoA ligase (4CL), chalcone isomerase (CHI), and phenylalanine ammonia lyase (PAL) and expression levels of PtPAL1 and Pt4CL were induced by *Funneliformis mosseae* inoculation under water stress, and AMF plants showed higher scavenging activity of hydroxyl radical and superoxide (O_2_^−^) by root flavonoid extracts under water stress, together with lower levels of O_2_^−^ and hydrogen peroxide and the degree of membrane lipid peroxidation in comparison with non-AMF plants, as well as increased flavonoid synthesis of trifoliate orange for reducing oxidative damage under water stress [230]. The single inoculation of *Claroideoglomus etunicatum* and *Rhizophagus clarus* promoted the accumulation of nitrogen in the aerial part of cowpea cultivars, and plants inoculated with *C. etunicatum* had similar or superior responses to plants fertilized with NPK or P [231]. Wheat inoculated with *Glomus intraradices* had maximum wheat yield and growth, which mechanically resulted from a higher rhizosphere colonization level, photosynthetic rate, and water use efficiency under drought stress [232]. 

Fungal inoculations partly improved fruit quality and mineral element components, depending on the fungi species, while the cultured mycorrhiza-like fungus *Piriformospora indica* relatively replaced AMF in citrus plants [233]. Inoculation with AMF provided good dry weight gain in lemon balm (*Melissa officinalis* L.), and notably contributed to high essential oil yield [234]. The mixed AMF inoculation in chamomile cultivation promoted both plant productivity and the quality of flower heads, especially regarding the component of phenolic compounds [235]. AMF inoculation had a positive effect on the yield of raspberry (*Rubus idaeus* L.) [236], while the combined implementation of biochar with AMF improved the colonization potential of AMF and significantly increased the photosynthetic potential of *Tamarindus indica* by increasing the contents of chlorophyll and carotenoids [237]. Different species of filamentous endophytic fungi, such as *Trichoderma*, are capable of controlling the pathogens *Xylella fastidiosa* and *Pseudomonas savastanoi* via the production and release of secondary metabolites; they are also effective against *Oomycetes* sp. and *Colletotrichum* sp. [238]. The combined application of GA_3_ (Gibberellic acid) and AMF (*Rhizophagus irregularis*) decreased growth impairment under salinity conditions by upregulating the hormonal balance of plants. The utilization of AMF was able to increase the productivity of sweet basil (*Ocimum basilicum*) plants under salinity conditions, and mycorrhizal inoculation significantly promoted water use efficiency and chlorophyll content under salinity stress [239]. AMF formation significantly boosted the high temperature tolerance of lettuce, a finding that could be related to the PSII system’s protection from damage under high temperatures [240]. Furthermore, it has been suggested that mycorrhizal symbiosis decreased the Na^+^ and Cl^−^ content and improved the relative water content (RWC), the total fresh and dry weight, and the photosynthetic activity of olive plants [241]. Mango (*Mangifera indica* L.) root stocks reacted to AMF inoculation in the nursery and also in the field with improved nutrient uptake, plant growth, and yield [242]. 

Rouphael et al. [243] concluded that the improvement in the biomass of crops after the application of two beneficial fungi, namely arbuscular mycorrhizal fungi and *Trichoderma koningii* TK7, could be connected to the modulation of the multilayer phytohormone interaction network, as well as a potential improvement in nitrogen use effectiveness via the glutamine oxoglutarate aminotransferase (GS-GOCAT) system. Hashem et al. [244] reported that the negative effects of salt stress in cucumber were ameliorated by AMF inoculation, which improved the activity of antioxidant enzymes that scavenged ROS and protected plant tissues from dehydration stress, including ascorbate peroxidase, catalase, and superoxide dismutase, and enhanced plant biomass and the synthesis of proline, pigments, and glycine betaine. Shekoofeh et al. [245] reported that AMF inoculation protected *Ocimum basilicum* plants from salinity stress by promoting water use efficiency, and increased chlorophyll synthesis and mineral uptake. Other examples of modes of action of AMF include the increased antioxidant activity and the accumulation of osmolytes [246], the adjusting of proline biosynthesis [246,247], and the accumulation of K, Ca, and Mg, which increased chlorophyll production and promoted the activity of enzymes [248,249]. Regarding the mitigating impacts of AMF against salinity stress, Estrada et al. [250] concluded that AMF inoculation restricted both uptake and accumulation of Na by adjusting the expression levels of SOS1, AKT2, and SKOR genes in roots, which allowed them to retain the homeostasis of Na^+^ and K^+^. The recent findings in omics science have also helped show that microbial biostimulants’ application involves great alterations in secondary and primary metabolites such as amino acids, phenolic acids, lipids, and tricarboxylic acid (TCA) intermediates, as well as alterations in protective mechanisms against stress that involve redox homeostasis, the stabilization of cell membranes, osmoregulation, the production of energy via amino acid degradation, and the increased expression of stress-related genes [150,251]. Qiu et al. [252] reported that the mitigation impacts of AMF increased with increasing AMF root colonization rate, and the mitigation impacts of AMF reduced with increasing soil P and N availability. Hu et al. [253] found that the symbiotic relationship between plant roots and AMF could affect the translocation and accumulation of pharmaceuticals and personal care products (PPCPs) in plants, and that substrate type can affect the function. 

The application of humic substances and AMF treatment led to a reduction in carvone content compared to treatment with humic substances alone, and it was suggested that AMF inoculation combined with a high P rate promoted essential oil content, whereas AMF inoculation combined with a high P rate and humic substance addition decreased the carvone content in *Lippia alba* (Mill.) N.E.Br. [254]. AMF can shorten the litter nutrient cycle, which is of great significance for the absorption, storage, and utilization of plant nutrients in the Songnen grassland ecosystem [255]. It has been reported that mycorrhizal colonization increased apple drought tolerance by improving gas exchange capacity, promoting chlorophyll fluorescence parameters, increasing scavenging of reactive oxygen species, creating a greater osmotic adjustment capacity, and using mitogen-activated protein kinase (MAPK) signals for interactions between AMF and their apple plant hosts [256]. Wang et al. [257] showed that AMF increased soil N_2_O emissions from legume systems by increasing P acquisition for biological N_2_ fixation, suggesting that AMF benefit crop production for higher yield and fewer N_2_O emissions in legume systems. The AMF *Gigaspora margarita* KKU-BH-01 can effectively increase the growth and aid eucalyptus seedlings to survive leaf blight disease (*Cylindrocladium quinqueseptatum*), as AMF induced plant chitinase and β-1,3-glucanase enzymes, resulting in improved disease resistance in plants [258]. Mycorrhizal oregano and mint plants had higher dry weight and essential oils, and the *Glomus etunicatum* strains were more effective than the *Glomus lamellosum* strain, and mycorrhizal oregano plants had higher dry weight than mycorrhizal mint plants [259]. Lam and Lai [260] reported that AMF treatment increased water spinach (*Ipomoea aquatica* Forsk.) growth and reduced the accumulation of Ni but not Cd. *Glomus mosseae* and *Glomus deserticola* increased shoot length, dry weight, chlorophyll quantity, and total N, P, and K components in *Eucalyptus globulus* shoots [261]. Gheisari Zardak et al. [262] concluded that the application of AMF could be important in the cultivation of medicinal plants under semi-arid and arid conditions. The most important impacts of AMF on different plants are shown in Table 1.

## 7. Modes of Action of Arbuscular Mycorrhizal Fungi (AMF)

AMF inoculation changed the biochemical procedures of lemongrass with a subsequent effect on growth and increased the suppression of *Fusarium* root rot under greenhouse conditions; additionally, essential oils (EOs) from mycorrhizal lemongrass frequently inhibited *Fusarium Solani* Fs4 development, notably more than the non-inoculated counterpart [389]. AMF can form a symbiosis with most vegetable plants, including the main crops from diverse families, such as Apiaceae (e.g., carrot), Amaryllidaceae (e.g., onion, garlic, leek), Cucurbitaceae (cucumber), Asteraceae (e.g., lettuce), Fabaceae (e.g., pea and bean), and Solanaceae (e.g., bell pepper, tomato) [390]. The most significant beneficial impacts of AMF are the boosted capture of mineral elements such as calcium, phosphorus, copper, zinc, sulfur, and iron, and the increased resistance to various types of abiotic and biotic stress [391,392]. In one experiment, it was reported that the α-diversity of AMF and diazotrophs communities, N-related enzymes, and sediment nutrition constituents were the principle parameters driving the biological nitrogen fixation (BNF) process in mangrove ecosystems [393]. The synchronous utilization of chitosan nanoparticles (CSNPs) and AMF promoted the levels of carotenoids, polyphenols, and tocopherols in the roots, thus increasing antioxidant capacity (33%); therefore, CSNPs can be used as effective biofertilization tools to increase plant growth and fitness, exemplified by the improvement in the health-promoting components in wheat [394]. In the presence of AMF inoculation strains *Rhizophagus irregularis* and *Funneliformis mosseae*, phosphorous components in the leaves of tomato plants were increased under both watering regimes, and even under limited watering conditions, Ca, K, Zn, Mg, and Mn were increased to levels similar to those of non-stressed plants [395]. AMF increased nutrient uptake, particularly phosphorus and other less mobile elements, mostly by increasing the absorptive surface of hyphal exploration in the rhizosphere, and acted as soil reclamation and biocontrol agents for contaminated or polluted soil [396]. Sarkar et al. [397] reported the probability of using arbuscular mycorrhizal fungi on symbiotic *Miscanthus* plants in phytoremediation and growing them in Zn-deficient soils. AMF improved plant growth under toxic trace element (TE) stress. The function of AMF in regulating heavy metal transport is concentration-dependent, and the transport direction of Cd and Zn in plants regulated by AMF changes with TE. AMF increased the absorption of Zn by growth dilution and Cd by biological enrichment, and the interaction between Cd and Zn was affected by AMF and other components [398]. 

Mycorrhizal fungi respond to metal contamination in different ways. *Funneliformis mosseae* in pepper can promote the dry mass production photosynthesis rate under Cu stress [399], *Glomus versiforme* and *Rhizophagus intraradices* can increase P nutrition and antioxidant activity of *Lotus japonica* under Cd stress [400], *Rhizophagus intraradices* can accumulate Cr in roots and decrease its translocation towards shoots of *Brachiaria mutica* under Cr stress, *Rhizophagus irregularis* may immobilize Cu and Pb in soil for the cultivation of willows [401], *Rhizophagus intraradices* can regulate As transporter in root epidermis cells and decrease its uptake in pea seedlings [402], and *Claroideoglomus* and *Rhizophagus* spp. can decrease Cd accumulation in wheat grains [403]. *Funneliformis mosseae* is a famous mycorrhiza among different AMF species for its symbiosis with the roots of various plant species, and it may facilitate the exchange of nutrients via the expansion of roots through the outer membrane of the mycelia and by boosting root development [404]. Some of the most important examples of mycorrhizal fungi mitigating soil nutrient deficiencies are *Rhizophagus irregularis* reducing soil nutrient deficiencies of P and Zn in *Medicago truncatula* by increasing *MtZIP5* and *MtPT4* gene induction [405], *Rhizophagus irregularis* boosting root absorption area and soil P availability in maize, *Rhizophagus irregularis* modifying the ZIP transporter response in barley and boosting grain Zn bioavailability [406], *Funneliformis mosseae* promoting the uptake of nutrients such as Mn, Mg, Zn, Fe, Ca, S, N, P, and K in cucumber [407], and *Rhizophagus irregularis* stimulating MtZIP6 gene expression and boosting the root absorption area in *Medicago truncatula* [406]. Various biochemical and physical mechanisms have been reported to play important roles in the enhancement of common bean plant stress resistance by AMF inoculation against *Rhizoctonia solani* or *Rhizoctonia* root rot disease, namely improved plant growth, increased plant nutrition, cytoplasmic granulation, improved cell wall thickening, and the accumulation of some antimicrobial components such as defense-related enzymes and phenolic substances [408]. Li et al. [409] reported that AMF promoted the accumulation of Cd in the roots of wheat but significantly decreased the amount of Cd in the grains and shoots. Moreover, stomatal conductance, photosynthetic rates, chlorophyll content, transpiration rates, and the accumulation of carbohydrates under Cd stress were elevated by AMF symbiosis. Proteomic analysis showed that AMF led to the expression of two enzymes that have significant functions in the chlorophyll biosynthesis pathway, namely Mg-protoporphyrin IX chelates and coproporphyrinogen oxide; increased the expression of two proteins related to CO_2_ assimilation, namely malic enzyme and ribulose-1,5-bisphosphate carboxylase; and boosted the expression of S-adenosylmethionine synthase. Under salinity stress, *glomus mosseae* can increase total proline and phenol content, leaf area, and root and shoot dry weights and lengths in *Triticum aestivum* [410]; *Funneliformis mosseae* can increase nutrient uptake, the photosynthetic rate, and the stand establishment rate of *Cucumis sativus* [411]; and *Rhizobium irregularis* and *Funneliformis mosseae* can increase plant biomass and decrease oxidative burst by strengthening the antioxidant enzymatic activities of *Cajanus cajan* [412]. Yu et al. [413] reported that AMF have ecological functions in decreasing Cd loss, with positive effects on maize growth because of leaching from polluted soil, as the AMF changed Cd migration by boosting the contents of glomalin-related soil protein (GRSP), exudates, and root morphology. Selvaraj et al. [414] observed that *Glomus intraradices* can improve both nutrient use efficiency and increase the defense response against herbivorous insects (*Spodoptera litura*) in blackgram. AMF symbiosis reduces temperature stress in host plants by improving the osmotic adjustment [415], increasing the photochemistry of PSII and the photosynthetic rate [416], increasing the uptake of nutrients [417], improving reproductive capacity, and enhancing antioxidant activity [418,419]. Farghaly et al. [420] reported that AMF inoculation can decrease the adverse impacts of alkalinity stress on wheat plants by decreasing Na concentration and pH and improving the availability of P and the productivity of wheat yield. 

## 8. Practical Application of Microbial Biostimulants on Crops

Culture-dependent approaches have allowed the isolation of various bacteria taxa from the mycorrhizosphere of *Glomus margarita*, *Glomus versiforme*, *Rhizobium irregular*, *Rhizobium clarus*, and *Funneliformis mosseae* [421,422,423]. Microbial inoculants in horticulture and agriculture systems have different effects; for example, *Pseudomonas putida* can increase iron uptake in rice, and *Pseudomonas fluorescens* can improve grain yield, plant height, and biomass in rice, sweet potato, and rapeseed [424,425,426]. The *Streptomyces* strain shows positive effects on the plant growth of tomato and rice [427,428]. Improved dry shoot weight and a significant increase in leaf length were reported after the application of *Azospirillum brasilense* Sp245, and *Aeromonas* spp. increased the root area of rice [429]. *Comamonas acidovorans* and *Bacillus subtilis* have significantly improved plant growth and increased cytokinin constituents in shoots and roots [430,431]. *Bacillus licheniformis* application increased fresh weight, enhanced cell division, and raised the chlorophyll content in cucumber [432], and *Azospirillum lipoferum* improved the root hair density of maize seedlings [433]. *Azospirillum lipoferum* increased tolerance to salinity stress in wheat [434], and *Pseudomonas putida* application led to increased shoot and root biomass and water content of white clover [435]. The utilization of *Alternaria* sp. stimulated drought tolerance in wheat [436,437]. It has also been reported that *Azoarcus* sp. increased plant nitrogen nutrition and root growth and reduced nutrient deficiency in wheat [438], and *Azorhizobium* sp. boosted plant nitrogen nutrition and root growth and alleviated nutrient deficiency [438]. *Rhizobium meliloti* improved plant growth, nitrogen use efficiency, and the quality of pods of peanuts [439,440], and *Rhizobium leguminosarum* boosted growth and yield performance under drought stress in soybean seedlings [441]. *Azotobacter chroococcum* and *Azotobacter vinelandii* increase root and shoot length, chlorophyll content, and root and leaf number [442]. Application of *Bacillus halotolerans, Pseudomonas frederiksbergensis RG2*, and *Enterobacter hormaechei* increased germination, growth, and yield; induced better drought resistance; and improved uptake of P, N, and Zn [443]. *Rhizoglomus irregulare* Aoufous consortium enhanced growth traits and physiological characteristics that have functions in the absorption of N and P content, increased protein and sugar content, reduced MDA and H_2_O_2_, alleviated soil pH, and increased the electrical conductivity, organic matter, and total organic carbon of date palm under drought conditions [444]. *Glomus* spp. increased root fresh weigh, root length, shoot height, shoot fresh weight, and germination index and improved the root basal diameter, dry biomass, and seedling length of date palm under heavy metal stress conditions [445]. AMF also can interact with PGPR [446,447]; for example, a positive and significant synergistic interaction has been reported between *Bacillus subtilis* and AMF regarding nitrate, nitrate reductase, and nitrogenase properties and the components of phenols, lipids, fiber, and osmoprotectants such as proline, betaine, and glycine [448]. Zamljen et al. [449] reported that microbial biostimulants added to the nutrient solution showed better results in the volatile content as well as primary metabolism and yield of basil. Anton-Herrero et al. [450] concluded that the origin and composition of microbial biostimulants determine their physiological impacts on pepper plants. Microbial biostimulants increased lipophilic antioxidants and yield and reduced sugars in cherry tomato cultivars [451,452,453,454,455,456,457,458,459,460,461,462,463]. 

## 9. Conclusions and Future Remarks

Biostimulants are sustainable and eco-friendly products that may promote metabolic and enzymatic processes in plants to enhance their yield, productivity, and crop quality and increase resistance to abiotic stresses. Biostimulants contain substances or products containing special formulations, natural components, or microorganisms that can naturally improve plant growth. The most important categories of biostimulants are humic and fulvic acid, fungi, bacteria, amino-acid-containing products, hormone-containing products, etc. Biostimulants are not only considered important alternatives to mineral fertilizers, but are also distinguished in organic farming systems under sustainable crop production management. Increased shoot and root growth, better root growth potential, improved resistance to stressors, and reduced nitrogen fertilizer inputs are some of the most noteworthy effects of biostimulant application in sustainable agriculture systems. 

Arbuscular mycorrhizal fungi are widespread root colonization fungi associated with the roots of higher plants. The current multiplication methods of mycorrhizal species under root organ culture (ROC) have become efficient alternatives for the cultivation of specific secondary metabolite compounds. Several kinds of studies have shown that AMF lead to significant changes in the quality and quantity of secondary metabolites that originate from medicinal and aromatic plants of economic interest. AMF can only be grown in the presence of host plants and are extensively used in agriculture and horticulture, especially *Rhizophagus* (formerly known as *Glomus*) *intraradices* and *Funneliformis* (formerly known as *Glomus*) *mosseae*. AMF make a positive contribution to growth, yield, stress tolerance, pathogen protection, and the maintenance of agricultural ecosystem sustainability. The increased activities of secondary metabolite production and antioxidant defense system enzymes related to plant defense have been extensively reported in mycorrhizal plants. 

In conclusion, AMF play a key role in plant nutrition and performance due to their capacity to improve plant mineral uptake. AMF, like other microbial biostimulants, can increase the sustainability of agricultural and horticultural production systems as well as improve the quality and quantity of food for the ever-growing world population. Moreover, studying the molecular mechanisms behind the observed activities will help to reveal the physiological and plant metabolism pathways involved in these processes and provide farmers with tailor-made products suitable for various conditions. However, further studies are needed to improve the reproducibility of the positive effects and to standardize the production processes from the lab to an industrial scale. This review article concludes that inoculation with specific arbuscular mycorrhizal fungi can increase the concentration of secondary metabolites in plants. However, future studies are needed to increase the reproducibility of the positive impacts, and more in-depth research is needed to identify the influence of interactions between AMF and biostimulants for promoting plants’ physiological characteristics. 

## Figures and Tables

**Table 1:** The most important effects of AMF on plants.

Type	Plant	Plant Family	Effects	Reference
Arbuscular Mycorrhizal Fungi (AMF)				
	Alfalfa (*Medicago sativa* L.)	Fabaceae	AMF inoculation may increase photosynthetic rates and the content of sugars in leaves.	[263]
	Asparagus (*Asparagus officinalis* L.)	Asparagaceae	AMF improved P efficiency via increased P uptake and optimal growth by adding AMF to suitable P fertilization.	[264]
	Asparagus (*Asparagus officinalis* L.)	Asparagaceae	AMF inoculation can enhance adaptation to salinity stress.	[265]
	Barrel medic (*Medicago truncatula* Gaertn.)	Fabaceae	AMF inoculation may alleviate Pb toxicity by improving the transport of sucrose from shoot to root, increasing the cleaving sucrose in roots, and increasing minor amino acid accumulation.	[266]
	Barrel medic (*Medicago truncatula* Gaertn.)	Fabaceae	AMF in combination with K can play an important role in reducing radiocesium uptake and its subsequent translocation to plant shoots.	[267]
	Basket willow (*Salix viminalis* L.)	Salicaceae	Willow AMF promoted the dissipation of soil polycyclic aromatic hydrocarbons (PAHs). Willow AMF increased the content of organic acids beneficial to metabolizing PAHs.	[268]
	Bishop’s flower (*Ammi majus*)	Apiaceae	Its application can induce the accumulation of phyto-molecules, coumarin, which might increase its medicinal and pharmacological applications.	[269]
	Black cumin (*Nigella sativa* Linn.)	Ranunculaceae	Colonization can increase relative water content (RWC), Chl b content, and micronutrient uptake.	[270]
	Black locust (*Robinia pseudoacacia* L.)	Fabaceae	AMF associated with black locusts may be useful to be used for improving the phytoremediation of Cd under elevated CO_2_ (ECO_2_).	[271]
	Cacao (*Theobroma cacao* L.)	Malvaceae	It can improve the overall growth and can positively increase the yield of cacao plants in acidic soils.	[272]
	Carob tree (*Ceratonia siliqua* L.)	Fabaceae	The used AMF *inocula* stimulated significantly the height of carob tree as well as the aerial dry weight.	[273]
	Castor bean (*Ricinus communis* L.)	Euphorbiaceae	AMF could protect castor bean against drought and salt stress by improving leaf exchanges and photosynthetic capacity and altering concentrations of metabolites.	[274]
	Chickpea (*Cicer arietinum* L.)	Fabaceae	AMF inoculation increased the final yield of chickpea.	[275]
	Cocoa (*Theobroma cacao* L.)	Malvaceae	AMF application improved the physical (cell wall turgor, root growth) and biochemical (proline, polyamines, enzymatic) characteristics of cocoa seedlings to reduce water stress.	[276]
	Durum wheat (*Triticum turgidum* subsp. *durum* (Desf.))	Gramineae	AMF can decrease water deficiency in cultivars, resulting in the up- and downregulation of many amino acids, phenylpropanoids, lipids, alkaloids, and hormones.	[277]
	Fenugreek (*Trigonella foenum*-*graecum* L.)	Fabaceae	AMF inoculation was effective in improving the tolerance of fenugreek to salinity.	[278]
	Fig (*Ficus carica* L.)	Moraceae	Fig was positively responsiveness to mycorrhizal inoculation, and the AMF induced different root architecture models.	[279]
	Foxtail millet (*Setaria italica*)	Poaceae	AMF application can decrease heavy metal phytoavailability and post-harvest transfer risks.	[280]
	Ginseng (*Panax quinquefolius* L.)	Araliaceae	AMF inoculation can promote plant uptake of N and P by suppressing soil-borne pathogens.	[281]
	Italian senna (*Cassia italica* Mill.)	Fabaceae	AMF inoculation increased the chlorophyll, protein, proline, and phenol content and lipid peroxidation.	[282]
	Lemongrass (*Cymbopogon citratus*)	Gramineae	AMF inoculation modified lemongrass metabolism with consequences on the essential oil component, composition, and antioxidant properties during growth.	[283]
	Maize (*Zea mays* L.)	Gramineae	Inoculation with AMF affects plant P constituent with or without P fertilizers addition. Inoculation may improve P availability in P-unamended and -amended soil.	[284]
	Myrrh (*Commiphora myrrha*)	Burseraceae	Mycorrhizal seedlings had higher biomass than non-mycorrhizal seedlings. Mycorrhizal seedlings had higher nutrient concentrations than non-mycorrhizal seedlings.	[285]
	Nemesia (*Nemesia* × *hybridus*)	Scrophulariaceae	AMF inoculation can improve flower yield and growth quality of nemesia. AMF can increase the response of the plant to irrigation with treated wastewater and reduce the cost associated with using other water sources.	[286]
	Nitre-bush (*Nitraria tangutorum* Bobr.)	Zygophyllaceae	The combination of AMF and PGPR significantly increased mycorrhizal colonization, promoted biomass accumulation, boosted morphological development, and improved photosynthetic performance, stomatal adjustment ability, and the exchange of water and gas.	[287]
	Oregano (*Origanum vulgare*)	Lamiaceae	The synchronous application of AMF and atmospheric CO_2_ (eCO_2_) promoted the accumulation of the majority of the detected sugars, amino acids, organic acids, phenolic acids, unsaturated fatty acids, and flavonoids. Both AMF and eCO_2_ treatments significantly promoted the growth and photosynthesis of oregano plants. Both AMF and eCO_2_ acted synergistically in improving the antioxidant capacity and anti-lipid peroxidation activity of oregano.	[288]
	Rice (*Oryza sativa* L.)	Gramineae	AMF could inhibit the uptake of Hg, particularly methyl-Hg in grains of rice. AMF caused Hg transfer in the non-edible parts of rice, such as leaf and stem. AMF improved the growth index and micro-indexes of rice.	[289]
	Rice (*Oryza sativa* L.)	Gramineae	The biochar combined with AMF improved soil nutrient availability and root growth strategy, and then promoted the nutrient absorption capacity of rice.	[290]
	Rice (*Oryza sativa* L.)	Gramineae	AMF may have a significant function in wetlands.	[291]
	Ryegrass (*Lolium multiflorum*)	Gramineae	AMF had a positive influence on the plant shoot biomass and the contents of P, N, Ca, K, and Mg in plants.	[292]
	Prickly pear (*Opuntia ficus*-*indica*)	Cactaceae	AMF promoted physiological and biochemical factors, and led to a decline in malondialdehyde (MDA) and hydrogen peroxide (H_2_O_2_).	[293]
	Seaberry (*Hippophae rhamnoides*)	Elaeagnaceae	AMF had a positive influence on the final yield.	[294]
	Sorghum (*Sorghum bicolor* L. Moench)	Gramineae	The total phenolic, carotenoid, flavonoid, and tannin concentrations were significantly higher in AMF–sorghum grain for all cultivars. The total phenolic, carotenoid, flavonoid, and tannin concentrations were significantly higher in AMF–sorghum grain for all cultivars.	[295]
	Sunflower (*Helianthus annuus* L.)	Asteraceae	AMF community has greater efficiency in promoting sunflower development and mycorrhizal colonization.	[296]
	Sunflower (*Helianthus annuus* L.)	Asteraceae	The combination of AMF and biochar increased antioxidant enzyme activity, nutrient content, osmoprotectants, and relative water content.	[297]
	Tomato (*Solanum lycopersicum* L.)	Solanaceae	AMF application has a significant influence on manganese, total nitrogen, and hydrophilic phenol components in the fruit.	[298]
	Wheat (*Triticum aestivum* L.)	Geramineae	AMF mitigated earthworm-induced N_2_O emissions from upland soil in a rice-rotated wheat farming system.	[299,300]
	Zucchini squash (*Cucurbita pepo* L.)	Cucurbitaceae	Inoculation may lead to better nutritional status of P, N, K, Mg, Ca, Zn, B, and Fe and low Al accumulation.	[301]
*Glomus mosseae*, *Glomus etunicatum*	Achnatherum (*Achnatherum sibricium* L.)	Gramineae	Simultaneous infections of both fungi significantly increased total phenolic concentrations.	[302]
*Glomus mosseae*	Alfalfa (*Medicago sativa* L.)	Fabaceae	It significantly enhanced Cd uptake by the roots of alfalfa under ET. It has no significant effect on iron-regulated transport 1 (*IRT1*) and natural resistance-associated macrophage protein 1 (*NRAMP1*) gene expression.	[303]
*Claroideoglomus etunicatum*	Alfalfa (*Medicago sativa* L.)	Fabaceae	The AMF alone or in combination with Si can alleviate salinity stress in alfalfa.	[304]
*Funneliformis mosseae*	Apple (*Malus domestica* Borkh.)	Rosaceae	The synergistic effect of dopamine and AMF improved apple salt resistance, and overexpression of MdTYDC promoted AMF symbiosis.	[305]
*Rhizophagus irregularis*	Barley (*Hordeum vulgare* L.)	Gramineae	The inoculation resulted in improved grain and straw Zn concentrations, especially at low soil Zn concentrations. AMF may be more appropriate for improving the quality of barley grain in terms of Zn concentrations, rather than improving yield.	[306]
*Rhizophagus intraradices*	Barrel medic (*Medicago truncatula* Gaertn.)	Fabaceae	Mycorrhizal colonization had little effect on root or shoot cesium (Cs) concentrations.	[307]
*Glomus caledonium*, *Glomus versiforme*	Bashfulgrass (*Mimosa pudica* L.)	Fabaceae	AMF inoculation significantly increased root mycorrhizal colonization rates and soil acid phosphate activities.	[308]
*Glomus mosseae*, *Gigaspora gigantea*	Carrot (*Daucus carota* L.)	Apiaceae	AMF inoculation can lead to successful carrot production under salinity stress.	[309]
*Glomus sinuosum*, *Paraglomus occultum*	Cassava (*Manihot esculenta Crantz*)	Euphorbiaceae	It can improve the final yield of cassava.	[310]
*Gloms rubiforme*, *Acaulospora scrobiculata*, *Glomus etunicatum*, *Glomus rubiforme*, *Acaulospora tuberculata*	Cassava (*Manihot esculenta Crantz*)	Euphorbiaceae	The inoculation had positive effects on cassava.	[311]
*Rhizophagus irregularis*, *Paraglomus* sp.	Castor bean (*Ricinus communis* L.)	Euphorbiaceae	Shoot Cr concentration doubled in non-AMF versus AMF plants; the content was similar. AMF vesicle percentage negatively correlated with Cr root concentration.	[312,313]
*Glomus tortuosum*	Chicory (*Cichorium intybus* L.)	Asteraceae	AMF, biochar, and N fertilizer application enhanced biomass.	[314]
*Glomus tortuosum*	Chicory (*Cichorium intybus* L.)	Asteraceae	AMF and biochar application increased nutrient absorption and reduced Cd absorption.	[314]
*Glomus clarum*	Chili (*Capsicum frutescens*)	Solanaceae	The inoculation increased the growth, flowering, and fruit production, and also increased the P uptake significantly.	[315]
*Glomus etunicatum*, *Funneliformis mosseae*	Cinnamomum (*Cinnamomum migao*)	Lauraceae	It markedly upregulated antioxidant enzyme activities and osmotic adjustment substances.	[316]
*Rhizophagus clarus*	Coarse Mint (*Mentha arvensis*)	Lamiaceae	Inoculation of coarse mint with AMF *Rhizophagus clarus* and a high dose of P boosted plant growth and the essential oil yield, and it increased carvacrol content.	[317]
*Acaulospora* sp., *Glomus* sp.	Common bean (*Phaseolus vulgaris* L.)	Fabaceae	The positive impact of co-infection by AMF and rhizobia on plant growth and the total N content of the plants was reported, along with a synergistic influence on the total P content, the number of nodules, and the mycorrhizal rate of the plants.	[318]
*Funneliformis mosseae*, *Rhizophagus irregularis*	Common myrtle (*Myrtus communis*)	Myrtaceae	AMF boosted myrtle drought resistance through enhanced water and nutrient supply and stimulation of antioxidant defense.	[319]
*Dominikia disticha*, *Claroideoglomus etunicatum*, *Rhizophagus irregularis*	Cowpea (*Vigna unguiculata* (L.) Walp.)	Fabaceae	Inoculation with all AMF led to high aboveground biomass production and accumulation of N as well as increased P content in plants.	[320]
*Glomus* Spp.	Cowpea (*Vigna unguiculata* (L.) Walp.)	Fabaceae	The activity of AMF in alleviating Cd stress in pre-flowering cowpea has been proven.	[321]
*Funneliformis mosseae*	Cucumber (*Cucumis sativus* L.)	Cucurbitaceae	The enhanced secondary metabolism and integrated transcriptional regulation might play a crucial role in AMF-mediated alleviation of chilling stress in plants.	[322]
*Glomus etunicatum*, *Glomus mosseae*, *Glomus versiforme*, *Glomus margarita*	Cucumber (*Cucumis sativus* L.)	Cucurbitaceae	AMF communities increased plant growth, soluble sugar content, chlorophyll content, and root activity.	[323]
*Glomus* spp., *Acaulospora* spp.	Cucumber (*Cucumis sativus* L.)	Cucurbitaceae	The AMF consortium could inhibit *Fusarium* wilt of cucumber, and, consequently, showed promising results as a biological control factor in greenhouse agro-ecosystems.	[324]
*Pervetustus simplex*, *Claroideoglomus etunicatum*, *Albahypha drummondii*, *Septoglomus xanthium*, *Funneliformis mosseae*, and *Rhizoglomus irregulare*	Date palm (*Phoenix dactylifera* L.)	Arecaceae	Shoot length and stem diameter were significantly higher in treatments augmented with compost and AMF.	[325]
*Glomus monosporus*, *Glomus deserticola*, *Glomus clarum*	Date palm (*Phoenix dactylifera* L.)	Arecaceae	All fungi significantly stimulated shoot height and biomass and increased the number of leaves per plant.	[326]
*Glomus iranicum*	Date palm (*Phoenix dactylifera* L.)	Arecaceae	It showed increased biomass production, chlorophyll, and mineral nutrient content.	[327]
*Claroideoglomus etunicatum*, *Rhizoglomus irregulare*, *Diversispora versiformis*	Eggplant (*Solanum melongena* L.)	Solanaceae	The inoculation is an effective strategy for alleviating cold stress.	[328]
*Rhizoglomus irregulare*	Eggplant (*Solanum melongena* L.)	Solanaceae	AMF improved fruit quality by reducing glycoalkaloid concentration and fruit browning potential.	[329]
*Gigaspora* gigantean, *Glomus mosseae*	Eggplant (*Solanum melongena* L.)	Solanaceae	Two mycorrhiza fungi affected plant growth indirectly, and in some situations, they reduced the inputs of chemical pesticides in eggplant.	[330]
*Glomus mosseae*	Fennel (*Foeniculum vulgare*)	Apiaceae	The mycorrhiza and growth-promoting bacteria (*Azospirillum*) resulted in the highest yields, total carotenoids, and chlorophyll in fennel plants subjected to water deficit stress.	[331]
*Funneliformis mosseae*	Grapevines (*Vitis vinifera*)	Vitaceae	The introduction of *F. mosseae* through donor plants is a suitable field inoculation method for grapevines and can help them to better withstand heat waves.	[332]
*Rhizophagus irregularis*	Hemp (*Cannabis sativa* L.)	Cannabaceae	AMF increased the heavy metal tolerance of hemp, and they changed Cd chemical forms by changing the composition of low molecular weight organic acids, which in turn affected soil Cd bioavailability.	[333]
*Rhizophagus intraradices*	Holy Basil (*Ocimum tenuiflorum* L.)	Lamiaceae	The inoculation increased the productivity of holy basil and boosted the quality of the final products.	[334]
*Rhizophagus clarus*, *Claroideoglomus etunicatum*, *Azospirillum brasilense*	Lemon grass (*Cymbopogon citratus* (DC.) Stapf)	Graminaeae	It is concluded that inoculating lemongrass with AMF enhances plant growth and development and modifies the content and essential oil composition.	[335]
*Rhizophagus clarus*	Maize (*Zea mays* L.)	Graminaeae	A combination of AMF (*Rhizophagus clarus*) and PGPR (*Bacillus* sp.) could enhance ^33^P uptake in maize plants under soil water stress.	[336]
*Funneliformis mosseae*	Maize (*Zea mays* L.)	Graminaeae	AMF massively improved biomass.	[337]
*Glomus intraradices*	Maize (*Zea mays* L.)	Graminaeae	After inoculation, there was an increase in leaf and stem ratios but a decrease in ear ratios.	[338]
*Funneliformis mosseae*, *Claroideoglomus etunicatum*	Maize (*Zea mays* L.)	Graminaeae	The inoculation increased bacterial diversity, decreased the relative abundances of selenobacteria related to plant Se absorption, and improved bacterial network complexity in selenium (Se)(VI)-stressed soils.	[339]
*Glomus clarum, Glomus deserticola*	Maize (*Zea mays* L.)	Graminaeae	30 g of *G. clarum* and *G. deserticola* had biocontrol potential against *Fusarium verticillioides*.	[340]
*Glomus intraradices*, *Glomus constrictum*, *Glomus mosseae*	Marigold (*Tagetes erecta* L.)	Asteraceae	It can improve the capability of reactive oxygen species (ROS) scavenging and reduce Cd concentration in plants to alleviate Cd stress in marigolds.	[341]
*Glomus constrictum* Trappe	Marigold (*Tagetes erecta* L.)	Asteraceae	AMF affected the host plant positively in growth, pigments, phosphorous content, and flower quality, and thus alleviated the stress imposed by water deficiency.	[342]
*Funneliformis mosseae*, *Claroideoglomus etunicatum*	Moldavian balm (Moldavian dragonhead) (*Dracocephalum moldavica* L.)	Lamiaceae	Inoculation may increase growth parameters and salinity tolerance under all salinity levels.	[343]
*Glomus mosseae*, *Glomus intraradices*	Mulberry (*Morus alba* L.)	Moraceae	AMF species colonization increased P and N contents of seedlings.	[344]
*Glomus deserticola*, *Gigaspora margarita*	Olive (*Olea europaea* L.)	Oleaceae	Mycorrhizal symbiosis decreased Na^+^ and Cl^−^ content and improved RWC, dry and fresh weight, and photosynthetic activity.	[345]
*Rhizophagus irregularis*	Olive (*Olea europaea* L.)	Oleaceae	The inoculation exhibited better performance under drought, especially under partial root-zone drying (PRD) treatment.	[345]
*Rhizophagus irregularis* DAOM 197,198	Olive (*Olea europaea* L.)	Oleaceae	Its colonization with olive roots significantly reduced the deleterious effect of water deficit stress by upregulating the primary and secondary metabolism and preserving a high stem water potential level in olive plants.	[346]
*Glomus intraradices*	Olive (*Olea europaea* L.)	Oleaceae	The mycorrhizal inoculation played an important part in the attenuation of the impacts of sulfates contained in gypsum substrate on olive trees.	[347]
*Funneliformis mosseae*, *Funneliformis constrictum*, *Gigaspora margarita*, and *Rhizophagus irregularis*	Onion (*Allium cepa* L.)	Amryllidaceae	Application of AMF and *Trichoderma viride* to onion plants assisted their growth in nutrient-deficient soils amended with fish waste.	[348]
*Rhizophagus intraradices*	Palmarosa (*Cymbopogon maritinii* (Roxb.) Wats. Var. Motia Burk	Gramineae	It may influence palmarosa seedling emergence and growth under salinity conditions, and it is useful for health and significant seedling emergence.	[349]
*Glomus intraradices*, *Glomus mosseae*, *Glomus etunicatum*	Papaya (*Carica papaya* L.)	Caricaceae	Rhizobacteria and AMF acting together formed a mutualistic relationship that enhanced disease control against *Fusarium oxysporum* and stimulated growth in papaya.	[350]
*Gigaspora margarita*	Peanut (*Arachis hypogaea*)	Anacardiaceae	The inoculation significantly enhanced leaf K accumulation, drought resistance, and pod yield under drought stress.	[351]
*Glomus intraradices*, *Gigaspora margarita*	Pepper (*Capsicum annuum* L.)	Solanaceae	Inoculated plants had greater dry weight compared to non-inoculated plants. The inoculation can increase P absorbance and P uptake.	[352,353]
*Glomus mosseae*, *Acaulospora laevis*, *Glomus manihotis*, and a mixed AMF strain	Pomegranate (*Punica granatum* L.)	Punicaceae	Growth, physiological, and biochemical activities were effectively improved by bio-hardening.	[354]
*Funneliformis mossae*, *Rhizophagus intraradices*	Quince (*Cydonia oblonga* Mill.)	Rosaceae	Inoculation with AMF led to significant enhancements in shoot and root dry weight and leaf chlorophyll content.	[355]
Genera *Scutellospora*, *Acaulospora*, and *Glomus*	Red clover (*Trifolium pratense* L.)	Fabaceae	After inoculation, significant increases in monoterpenes such as myrcene, (-)-β-pinene, and linalool were observed.	[356]
*Glomus aggregatum*, *Funneliformis mosseae*, *Rhizophagus intraradices*	Red tangerine (*Citrus reticulata* Blanco)	Rutaceae	AMF had a positive effect on red tangerine.	[357]
*Claroideoglomus etunicatum*	Redtip (*Photinia fraseri* Dress)	Rosaceae	AMF had an impact on plant height and hyphal length density.	[358]
*Rhizophagus intraradices*	Rice (*Oryza sativa* L.)	Gramineae	It significantly reduced total As and inorganic As components in rice grains.	[359,360]
*Acaulospora mellea*; *Glomus formosanum*; *Rhizoglomus clarum*; *Glomus* spp.	Rice (*Oryza sativa* L.)	Gramineae	It stimulated plant growth, improved root morphological characteristics, and increased P accumulation in rice plants under salt stress conditions.	[361,362]
*Glomus* sp1	Rose (*Rosa rubiginosa* L.)	Rosaceae	The highest percentage of rose root stock establishment increment was achieved with the application of *Glomus* sp1.	[363]
*Glomus etunicatum*, *Glomus mosseae*	Ryegrass (*Lolium perenne*)	Gramineae	AMF-inoculated plants showed lower Cd toxicity, despite the increase in Cd uptake.	[364]
*Funneliformis mosseae*, *Claroideoglomus etunicatum*	Salt grass (*Puccinellia tenuiflora*)	Gramineae	It is able to alleviate boron (B) toxicity by improving biomass and reducing tissue B concentrations. It can help plants tolerate the combined stresses of salt and drought.	[365]
*Rhizophagus intraradices*, *Funneliformis mosseae*	Saffron (*Crocus sativus* L.)	Iridaceae	The mixture of both species increased the spice yield, quality, antioxidant activity, and bioactive compound contents.	[366]
*Glomus mosseae*	Snap bean (*Phaseolus vulgaris* L.)	Fabaceae	AMF increased the concentrations of P, N, Mg, and Ca in roots and shoots. It can be concluded that it may reduce the detrimental impacts of increasing O_3_ on host plants by improving plant nutrition and growth.	[367]
*Glomus mosseae*, *Glomus intraradices*, *Glomus hoi*	Sour orange (*Citrus aurantium* L.)	Rutaceae	Under salt stress, mycorrhizal-inoculated plants had higher chlorophyll content, higher growth, lower electrolyte leakage, better water status, greater gas exchange capacity, higher malondialdehyde and hydrogen peroxide content, higher osmolyte accumulation, and better antioxidant defense systems.	[368]
*Glomus mosseae*, *Glomus intraradices*	Sorghum (*Sorghum bicolor* L. Moench)	Gramineae	AMF can change the profile of VOCs emitted by roots as well as root morphology. AMF can positively affect the morphological traits of the host roots, total root length, and specific root length of mycorrhizal plants.	[369]
*Glomus* sp. 1, *Glomus* sp. 2, *Glomus* sp. 3, *Glomus aggregatum*, *Glomus fasciculatum*, *Acaulospora longula*, *Glomus occultum*, *Acaulospora scrobiculata*, *Acaulospora spinosa*, *Scutellospora* sp.	Sorghum (*Sorghum bicolor* L. Moench)	Gramineae	AMF application improved P and K uptake in shoots.	[370]
*Funneliformis mosseae*; *Funneliformis geosporum*	Sorghum	Gramineae	Plant height and fresh and dry biomass of AMF-inoculated plants were greater in normal soil, followed by sodic and saline soils.	[371]
*Acaulospora saccata*, *Acaulospora fragilissima*, *Scutellospora ovalis*, *Rhizophagus neocaledonicus*, *Claroideoglomus etunicatum* nc, *Pervetustus simplex* nc	Sorghum (*Sorghum bicolor* L. Moench)	Gramineae	Inoculum of combined AMF isolates is appropriate to obtain higher yields and less contaminated biomass of forage sorghum in ultramafic environments.	[372]
*Glomus mosseae*, *Rhizophagus irregularis*	Soybean (*Glycine max* L.)	Fabaceae	The inoculation can enhance P uptake and soybean productivity.	[373]
*Rhizophagus clarus*	Soybean (*Glycine max* L.)	Fabaceae	AMF inoculation positively influenced grain yield, shoot dry weight, and P and N content in leaves.	[374]
*Cetraspora pellucida*, *Claroideoglomus etunicatum*	Strawberry (*Fragaria* × *ananassa* Duch.)	Rosaceae	Plants grown with 9% biochar and inoculated with *C. etunicatum* showed a more profuse root system.	[375]
*Rhizophagus clarus*	Strawberry (*Fragaria* × *ananassa* Duch.)	Rosaceae	AMF significantly enhanced plant biomass production by boosting photosynthesis rate, antioxidant enzyme defense, water content and use efficiency, and the nutritional status of Zn, in particular.	[376]
*Rhizophagus intraradices*	Sweet flag (*Acorus calamus*)	Acoraceae	Under Cr stress, AMF promoted nutrient uptake by *A. calamus* and increased soil carbon input. AMF significantly increased the synergy between the dominant strains.	[377]
*Rhizophagus fasciculatus*, *Rhizophagus aggregatus*, *Rhizophagus irregularis*	Tangerine orchard (*Citrus reticulata* L.)	Rutaceae	Inoculation had a positive effect on the final yield.	[378]
*Glomus versiforme*	Tobacco (*Nicotiana tabacum* L.)	Solanaceae	AMF can protect tobacco against As uptake, and it can play an important role in food quality and safety.	[379]
*Claroideoglomus etunicatum*, *Claroideoglomus claroideum*, *Glomus microaggregatum*, *Rhizophagus intraradices*, *Funneliformis mosseae*, *Funneliformis geosporum*	Tomato (*Solanum lycopersicum* L.)	Solanaceae	Mycorrhizal inoculation significantly boosted root colonization levels, height, root dry biomass, total yield, shoot dry biomass, and number of fruits.	[380]
*Funneliformis Mosseae* and *Rhizophagus intraradices*	Thyme (*Thymus vulgaris* L.)	Lamiaceae	Their inoculation increased essential oil production in both *Thymus vulgaris* L. and *Thymus daenensis* under water stress conditions.	[381]
*Glomus versiforme*	Trifoliate orange (*Poncirus trifoliata*)	Rutaceae	Mycorrhization significantly increased gallic acid, ferulic acid, salicylic acid, and phlorizin acid.	[382]
*Rhizophagus intraradices*, *Funneliformis mosseae*	Valerian (*Valeriana officinalis* L.)	Caprifoliaceae	They considerably improved root proline and total soluble sugars and total phenolics in roots and shoots versus untreated valerian plants.	[383]
*Funneliformis mosseae*, *Glomus versiforme*	Wheat (*Triticum aestivum* L.)	Graminae	Inoculation with AMF could increase Se bioavailability in the rhizosphere.	[384]
*Funneliformis mosseae*, *Glomus versiforme*	Wheat (*Triticum aestivum* L.)	Graminae	AMF combined with 48.76 mgkg^−1^ P applied in soil can not only achieve high grain yield, but also fully exploit the biological potential of Se uptake in wheat.	[385]
*Funneliformis mosseae* BGC HEB02, *Rhizophagus intraradices* BGC HEB07D	Wheat (*Triticum aestivum* L.)	Graminae	Zn in wheat grain can be significantly increased by inoculation with AMF, indicating the potential of AMF to cope with Zn deficiency.	[386]
*Glomus mosseae*, *Glomus hoi*, *Glomus etunicatum*, *Acaulospora scrobiculata*, *Acaulospora spinosa*	White yam (*Dioscorea rotundata*)	Dioscoreaceae	AMF can increase yam tuber growth.	[387]
*Funneliformis mosseae*, *Laroideoglomus etunicatum*, *Rhizophagus intraradices*	Willow (*Salix viminalis*)	Salicaceae	Organic acids including arachidonic acid, octadecanedioic acid, α-linolenic acid, 10,12,14-octadecarachidonic acid, and 5-methoxysalicylic acid were significantly increased under AMF inoculation treatment. AMF inoculation also increased the levels of polyphenol oxidase and dehydrogenase.	[388]

## Data Availability

Not applicable.
